# Late Presentation of Xeroderma Pigmentosa With Squamous Cell Carcinoma in Septic Shock: Report of a Rare Case

**DOI:** 10.7759/cureus.25967

**Published:** 2022-06-15

**Authors:** Christopher J Pinto, Rajesh Nayyar, Dandamudi Asvita, Avinash Chirumamilla, Prachi Patel

**Affiliations:** 1 Medical Research, Karnataka Insititute of Medical Sciences, Hubballi, IND; 2 Internal Medicine, Washington University School of Medicine, Washington, USA; 3 Emergency Medicine, MediCiti Institute of Medical Sciences, Ghanpur, IND; 4 Infectious Diseases, Kanachur Institute of Medical Sciences, Natekal, IND; 5 Medicine, Tianjin Medical University, Tianjin, CHN

**Keywords:** pediatrics emergency, squamous cell neoplasm, septic shock in children, pediatric rare diseases, dermato-oncology, rare skin disease, xeroderma pigmentosum

## Abstract

Xeroderma pigmentosum (XP) is a rare autosomal recessive pathology affecting nucleotide excision repair against ultraviolet radiation. This leads to an increased predisposition to developing ophthalmological, neurological, and cutaneous conditions with an increased cell turnover. This case reports a late presentation of XP presenting with metastatic squamous cell carcinoma (SCC) and septic shock in an eight-year-old Indian male. Emergency management with IV fluid boluses and broad-spectrum antibiotics showed no improvement in vitals. Urgent surgical debridement and tumor debulking failed to improve laboratory values. Postoperative leukocytosis with fever spikes warranted the need to transfer the patient to a super-specialty oncology unit. Such an adverse presentation is commonly seen in XP-related invasive squamous cell carcinoma. Preventive management requires early identification and a multidisciplinary approach involving dermatologists, ophthalmologists, and surgeons. Late presentations revolve around control of the disease process by sharp debridement and chemotherapy with regular surveillance as the lesions tend to reoccur even after excision and chemotherapy.

## Introduction

Xeroderma pigmentosum (XP) is an autosomal recessive pathology affecting DNA repair against UV-induced thymine dimers and photoproducts [1]. Patients afflicted are known to have a 10,000-fold risk of developing non-melanoma skin cancer in comparison to the general population [1]. This is caused by defects in eight known complementation genes (*XPA* to *XPG* and XP variant) [1,2]. Patients with the *XPA, XPB, XPD, XPF*, and *XPG* variants have an exaggerated response to minimal exposure to sunlight [2]. Cutaneous manifestations are divided on the basis of onset. In response to sunlight, acute onset cutaneous manifestations may present with widespread erythema, blisters, and erosions. Late-onset cutaneous manifestations are a sign of actinic cell damage and increased cell turnover [1,3]. Lentiginous macules may further evolve into widespread xerosis and multiple malignant skin tumors [3]. Ocular manifestations could show xerosis, conjunctivitis, corneal scarring, conjunctival melanosis, and neoplastic ocular surface lesions [4]. Neuroimaging may show mild brain atrophy despite having normal cognitive function [5]. Patients may also have learning difficulties with diffuse encephalopathy followed by motor signs and cerebellar ataxia [3,6]. Most patients develop their first non-melanoma skin cancer at the age of nine years [1]. The most common cause of mortality in such patients is invasive squamous cell carcinoma or malignant melanoma with a median age of survival of less than 40 years of age [1].

Early recognition of the disease is based on the characteristic accentuation of burning sensation in addition to the appearance of hyperpigmented macules on exposure to sunlight. Patients need to receive life-long dermatologic surveillance and constant use of preventive barrier lotions/clothing. Patients though initially treated for squamous cell carcinoma with the help of wide local excision, the disease of XP itself renders the inability to form stable UV non-sensitive tissues [1]. And hence, recurrence rates of skin cancers despite surgery may push towards an aggressive form leading to a late presentation as seen in this titled case report [1,3].

## Case presentation

History

An eight-year-old boy presented as a referral to our Emergency Department in a government-run hospital in Hubballi, Karnataka, India, with complaints of copious foul discharge from multiple head lesions with a fever that began one month prior to presentation. From late infancy, multiple skin lesions were noted on the head, neck, and chest. These lesions had a burning characteristic accentuated by exposure to sunlight. Partial relief was noted with the application of moisturizers and UV skin protectors. The lesions were initially planar, which progressed over years to an erosive base with eruptive edges suggestive of squamous cell carcinoma. Treatment with wide excision and chemotherapy sessions was done one year prior to the current presentation requiring inpatient care for two months. Past history is significant for repeated visits with complaints of eye irritation and corneal ulceration.

A significant social history shows an agrarian impoverished background in a nuclear family of five. Family history is significant for parental second-degree consanguineous marriage. Past history is notable for diagnosed squamous cell carcinoma requiring surgical excision and four sessions of chemotherapy two years back. Due to paucity of monetary funds, parents opted for alternative non-allopathic therapy based on cultural and religious beliefs leading to the current late presentation.

Physical findings and systemic examination

**Figure 1:**
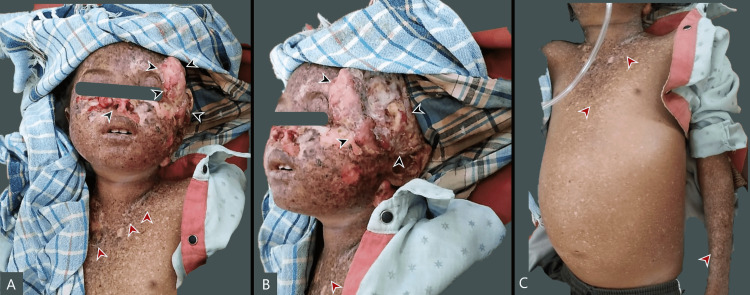
A. Anterior view of the face showing multiple erosive lentiginous macules (marked in red), large fungating polypoid mass (marked in black), and previously debrided tip of the nose (marked on green); B. Lateral view of the head showing a large fungating mass (marked in black) measuring 18cm x 15cm over the temporal region with thick exudates; C. Ascites with scattered pigmented lentiginous macules over the collar region and hands (marked in red)

Vitals of the child showed a heart rate of 134/minute and blood pressure of 72/52 mm Hg. Breathing rate was 38/min with the use of accessory muscles and oxygen saturation was 88% with a temperature of 39.8º Celsius (103.6º F). The child was not oriented to time, place, or person. Physical examination showed a cachexic child with multiple hyperpigmented lentiginous macules. The macules were distributed over areas of the upper chest, head, and neck regions. Multiple skin lesions with everted hypertrophied edges were noted over the head (Figures 1A, 1B). The base of the largest lesion was hard, with thick foul-smelling exudates from draining sinuses from the adjacent tissues. The maximum vertical extent of the ulcer was to the underlying temporalis muscle and the horizontal skin extent was from the temporal apex to the mid-ear region. Clinical features of all the lesions were consistent with recurrence of squamous cell carcinoma as an outcome of XP. Surgical excision of the tip of the nose was done at the previous visit in view of squamous cell carcinoma. Bilateral cervical subgroup lymph nodes were enlarged with multiple nodes measuring 3 cm, fixed and stony in consistency.

Cardiovascular system examination was consistent with septic shock-induced tachycardia. A respiratory system examination showed bilateral pulmonary rales. Abdominal examination showed a distended ascitic abdomen with positive fluid thrill (Figure 1C). Ocular examination showed dry corneal membranes and erythematous conjunctiva suggestive of xerosis.

Laboratory investigations

Blood investigations showed a hemoglobin of 7.2g/dl (N=12-16g/dl) with a white blood count of 42,000 cells/dl (N=3500-9000). Serum lactic acid levels were 16 mmol/L (N=<2mmol/L). Arterial blood analysis showed a blood pH of 7.23, with a bicarbonate (HCO_3_) level of 12 and partial pressure of carbon dioxide (pCO2) level of 29mmHg (N=35-45). Serum electrolyte levels showed sodium (Na^+^) at 130 meq/L (N=135-145 meq/L) and potassium (K^+^) level of 4.1 meq/L (N=3.5-5 meq/L). Renal function showed a raised creatinine level of 2.3mg/dl (N=06-1.2mg/dl).

Treatment and outcome

A clinical diagnosis of XP-induced metastatic squamous cell carcinoma with bacterial septic shock was made in view of history and physical findings. Immediate IV fluid resuscitation with bolus Ringer's lactate solution was started at the rate of 20ml/kg for the first 15 minutes. Titration of IV fluids at 10ml/kg every 30 minutes along with IV epinephrine at the dose of 0.1 mcg/kg/min was started as a continuous infusion. The patient was intubated in view of worsening shock and poor sensorium. The patient was also started on IV ceftriaxone, vancomycin, clindamycin, and acetaminophen.

**Figure 2:**
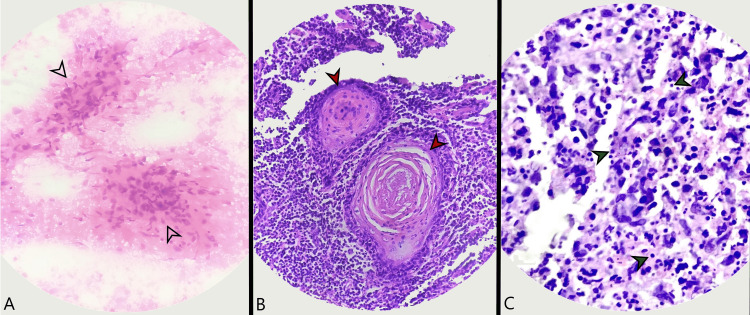
A. Exudate cell cytology showing dense neutrophilic aggregates (Gram stain); B. Postoperative specimen showing keratin whorls in a bed of neutrophilic infiltrate (H&E) (40x); C. Post-operative specimen showing gram-positive cocci (a few marked in green) with dense neutrophilic aggregates (H&E) (100x) H&E: hematoxylin and eosin

Swabs from the lesion were collected for cell cytology and culture and sensitivity (Figure 2A). The cultured pus grew mixed flora. An emergency opinion of a surgeon indicated the need for sharp debridement of the wet gangrenous tissue. The patient was taken up for tumor debulking and debridement. No intra-operative complications were noted. Multiple samples from multiple masses were sent for histopathology (Figures 2B, 2C). Postoperatively, the patient’s vitals showed no improvement. The patient was continued on broad-spectrum antibiotics, fluid therapy, and epinephrine under intensive care. Following intensive care, on day three of post-operative care, in light of persistent leukocytosis and a fever spike to 41.0º Celsius (105º F), the child was transferred to a government-run surgical-oncology super-specialty unit within the same state, in Bangalore, Karnataka, India (Table 1). The patient improved after 25 days of inpatient surgical-oncology care requiring skin grafts and was then placed on oral retinoids at the dose of 2 mg/kg. Post graft acceptance, the patient was scheduled follow-up chemotherapy sessions at our center.

**Table 1:** Blood investigations trend during the preoperative and postoperative period LAP: leukocyte alkaline phosphatase; WBC: white blood cell; PCO2: partial pressure of carbon dioxide

Investigations	Preoperative Day 1	Postoperative Day 1	Postoperative Day 2	Postoperative Day 3
WBC count	42,000	56,000	52,000	76,000
LAP score	216 (leukemoid reaction)	-	-	230 (leukemoid reaction)
Hemoglobin	7.2 > (1 unit transfused) > 9.1	8.8	8.9	8.6
Blood urea nitrogen	42	40	46	40
Creatinine	2.3	2	1.86	1.7
Lactic acid levels	16	14	12	14
Sodium	130	134	135	134
Potassium	4.1	4	4.1	3.8
Blood gas analysis				
Blood PH	7.23	7.38	-	7.32
Bicabonate	12	18	-	20
pCO2	29	31	-	39
Vitals				
Blood pressure	72/52	108/60	98/66	92/60
Pulse	134	110	90	112
Temperature	39.8 Celsius	39 Celsius (spikes to 41)	39.6 Celsius(spikes to 41)	39.3 Celsius (spikes to 40)
Respiratory rate	38	28	26	26
Oxygen saturation	88	95	94	91

## Discussion

XP was first mentioned in the 19th century by Moriz Kaposi, who described four patients with parchment-like skin with an abrupt line of demarcation [7,8]. In 1926, XP was understood to be extreme sensitization of the skin in response to ultraviolet radiation [7,9]. The pathologic mechanism in XP, an autosomal recessive disease, is that nucleotide excision repair (NER) against UV-induced thymine dimers and photoproducts are hampered [1]. This disease is known to be caused by defects in eight complementation genes (*XPA* to *XPG* and XP variant) [1,2]. Defects in each subtype have variable outcomes on UV sensitivity and neuro-psychiatric sequelae as seen in Table 2 [1,10,11].

**Table 2:** Xeroderma pigmentosum genes with features XP: xeroderma pigmentosum; UV: ultraviolet radiation

XP genes	Protein function	Defective pathway	Features
XPA	Damage verification	Nucleotide excision repair	Most common subtype in Japan, presents with moderate neurological dysfunction. Severe UV sensitivity.
XPB/ERCC3	Helicase		Features of XP with mild neurological dysfunction. Moderate UV sensitivity.
XPC	Damage recognition		Common subtype in United States, Africa, and Europe. Features of XP without any neurological dysfunction. Moderate UV sensitivity.
XPD/ERCC2	Helicase		Features of XP with moderate neurological dysfunction. Moderate UV sensitivity.
XPE/DDB2	Damage recognition		Mild XP features without any neurological dysfunction. Mild UV sensitivity
XPF/ERCC4	Nuclease		Second-most common subtype in the Japanese population. Mild features of XP with mild to no neurological dysfunction. Mild UV sensitivity
XPG/ERCC5	Nuclease		Features of XP with moderate neurological dysfunction. Moderate UV sensitivity.
XPV/POLH	Repair by translesion synthesis	Defective post-replication repair	Common subtype in United states, Africa and Europe. Mild XP features without any neurological dysfunction. No UV sensitivity.

The incidence of XP in Europe and the United States is one in every one million individuals and the Incidence in Japan is one in every 22,000 individuals [1]. The incidence in Libya is estimated to be one in every 5000 individuals due to the cultural norms enabling consanguinity [1,12]. Incidence of XP in the Indian population is unknown but is estimated to be slightly lower than the European prevalence [13]. The clinical features in XP involve mainly three systems: cutaneous, ocular, and neurological [3]. Acute cutaneous manifestations are seen due to extreme UV sensitivity causing exaggerated sunburns even with minimal exposure. Such sunburns often take a longer period to resolve, as seen in our patient, taking 7-10 days to heal [1]. Development of hyperpigmented lentiginous macules and hypopigmented patches, as seen in our patient, can occur before the age of two. Such changes are commonly seen along the sun-exposed regions of the hand, the nape of the neck, and the face. Due to actinic damage of the skin in response to UV light, the skin may undergo premature aging and atrophy. Long-term sequelae may include the development of basal cell carcinomas, invasive squamous cell carcinomas, and malignant melanomas. Most patients develop their first non-melanoma skin cancer at the age of nine years and first melanoma skin cancer at a median age of 22 years [1]. Squamous cell carcinomas seen herein are difficult to treat as they are fast-growing, invasive, and often metastasize [14]. The most common cause of mortality is invasive squamous cell carcinoma or malignant melanoma with a median age of survival of less than 40 years [1,15].

Ocular manifestations are seen in virtually every single XP patient with cutaneous findings [1,2,4]. Initial presentation of corneal irritation followed by progressive ulceration leading to pterygium, pannus formation, and permanent corneal scarring. Late ocular changes may show neoplastic ocular surface lesions [3,4]. Neurologic findings can occur before the age of eight and in some cases, neuroimaging may show mild brain atrophy despite having normal cognitive function [5,6]. Neurological sequelae are caused as pathways of oxidative damage repair in neuronal tissue follow a common pathway as seen in NER in response to UV [12]. However, such neurologic sequelae were not noted in our patient hinting toward a probable *XPC* gene involvement [1,2,10,11].

Management requires early identification and a multidisciplinary approach involving dermatologists, ophthalmologists, and surgeons. In this case, supportive management with IV fluids, pressors, and antibiotics was given in adjunct to surgical dissection. The cause of septic shock here was attributed to the disease process of skin cancer with secondary septicemia. This diagnosis was based on symptoms and age of presentation in aid with dermatological characteristic accentuation [13,16]. The absence of nasal alar cartilage was due to previous surgical excision over the tip of the nose instead of squamous cell carcinoma involvement. Genetic testing is recommended in such cases to identify the specific gene defect; however, since this was an emergency presentation, it could not be done. Follow-up surveillance (every three to six months) by comparison of photographs of sun-exposed regions may help the physician navigate a treatment course in early presentations. Lesions that show growth or erosion are to be biopsied for early diagnosis and identification of the type of skin cancer. Patients are recommended to regularly apply sunscreens with the use of sunlight barriers [1,17]. In late presentations of the disease, patients are to undergo extensive surgical consults and chemotherapy for metastatic lesions. In many cases, the lesions tend to reoccur even after excision and chemotherapy [17].

The use of retinoids in the treatment of XP-related skin cancers has been effective in doses ranging from 0.5mg/kg/day to 2mg/kg/day [3,17]. The use of recombinant liposomal encapsulated T4 endonuclease V and topical DNA repair enzymes in XP-related skin disorders have been proven to be useful but are expensive in practical usage [17,18]. Attempts with gene therapy by retrovirus-mediated gene transfer have shown promising results and could be a viable modality in the future to directly repair the affected XP genes [13,19].

## Conclusions

XP is a rare disease affecting cell repair against UV radiation. In our case, a late-stage presentation of XP in septic shock required intensive antibiotic therapy, tumor debulking, and skin grafting. Preventive management requires early identification and a multidisciplinary approach involving dermatologists, ophthalmologists, and surgeons. Late presentations revolve around control of the disease process by sharp debridement and chemotherapy with regular surveillance.

## References

[1] Lucero R, Horowitz D (2022). Xeroderma pigmentosum. StatPearls [Internet].

[2] Nishigori C, Nakano E, Masaki T, Ono R, Takeuchi S, Tsujimoto M, Ueda T (2019). Characteristics of xeroderma pigmentosum in Japan: lessons from two clinical surveys and measures for patient care. Photochem Photobiol.

[3] Kraemer KH, Lee MM, Scotto J (1987). Xeroderma pigmentosum. Cutaneous, ocular, and neurologic abnormalities in 830 published cases. Arch Dermatol.

[4] Brooks BP, Thompson AH, Bishop RJ (2013). Ocular manifestations of xeroderma pigmentosum: long-term follow-up highlights the role of DNA repair in protection from sun damage. Ophthalmology.

[5] Anttinen A, Koulu L, Nikoskelainen E (2008). Neurological symptoms and natural course of xeroderma pigmentosum. Brain.

[6] Taylor AM (2008). Neurodegeneration in xeroderma pigmentosum. Brain.

[7] DiGiovanna JJ, Kraemer KH (2012). Shining a light on xeroderma pigmentosum. J Invest Dermatol.

[8] Hebra F, Kaposi M (1874). Xeroderma. On diseases of the skin including exanthemata.

[9] Per M (1926). Xeroderma pigmentosum (Kaposi): Report of a case, with special reference to clinical features and pathogenesis. Br J Dermatol.

[10] Lehmann AR, McGibbon D, Stefanini M (2011). Xeroderma pigmentosum. Orphanet J Rare Dis.

[11] Abeti R, Zeitlberger A, Peelo C, Fassihi H, Sarkany RP, Lehmann AR, Giunti P (2019). Xeroderma pigmentosum: overview of pharmacology and novel therapeutic strategies for neurological symptoms. Br J Pharmacol.

[12] Hand JL, Warner CG (2022). Xeroderma pigmentosum. UpToDate.

[13] Mareddy S, Reddy J, Babu S, Balan P (2013). Xeroderma pigmentosum: man deprived of his right to light. ScientificWorldJournal.

[14] Halkud R, Shenoy AM, Naik SM, Chavan P, Sidappa KT, Biswas S (2014). Xeroderma pigmentosum: clinicopathological review of the multiple oculocutaneous malignancies and complications. Indian J Surg Oncol.

[15] Lopes-Cardoso C, Paes da Silva Ramos Fernandes LM, Ferreira-Rocha J, Teixeira-Soares C, Antônio-Barreto J, Humberto-Damante J (2012). Xeroderma Pigmentosum - a case report with oral implications. J Clin Exp Dent.

[16] (2022). Xeroderma Pigmentosum: Disease at a glance. https://rarediseases.info.nih.gov/diseases/7910/xeroderma-pigmentosum.

[17] Lambert WC, Lambert MW (2015). Development of effective skin cancer treatment and prevention in xeroderma pigmentosum. Photochem Photobiol.

[18] Herouy Y, Krutmann J, Norgauer J, Schöpf E (2003). Xeroderma pigmentosum: children of the moon (Article in German). J Dtsch Dermatol Ges.

[19] Dupuy A, Valton J, Leduc S (2013). Targeted gene therapy of xeroderma pigmentosum cells using meganuclease and TALEN™. PLoS One.

